# An Autocrine Proliferation Repressor Regulates *Dictyostelium discoideum* Proliferation and Chemorepulsion Using the G Protein-Coupled Receptor GrlH

**DOI:** 10.1128/mBio.02443-17

**Published:** 2018-02-13

**Authors:** Yu Tang, Yuantai Wu, Sarah E. Herlihy, Francisco J. Brito-Aleman, Jose H. Ting, Chris Janetopoulos, Richard H. Gomer

**Affiliations:** a Department of Biology, Texas A&M University, College Station, Texas, USA; b Department of Biological Sciences, University of the Sciences in Philadelphia, Philadelphia, Pennsylvania, USA; Cornell University

**Keywords:** cell density sensing, chemorepulsion, *Dictyostelium*, G-protein-coupled receptors, cell proliferation, quorum sensing

## Abstract

In eukaryotic microbes, little is known about signals that inhibit the proliferation of the cells that secrete the signal, and little is known about signals (chemorepellents) that cause cells to move away from the source of the signal. Autocrine proliferation repressor protein A (AprA) is a protein secreted by the eukaryotic microbe *Dictyostelium discoideum*. AprA is a chemorepellent for and inhibits the proliferation of *D. discoideum*. We previously found that cells sense AprA using G proteins, suggesting the existence of a G protein-coupled AprA receptor. To identify the AprA receptor, we screened mutants lacking putative G protein-coupled receptors. We found that, compared to the wild-type strain, cells lacking putative receptor GrlH (*grlH¯* cells) show rapid proliferation, do not have large numbers of cells moving away from the edges of colonies, are insensitive to AprA-induced proliferation inhibition and chemorepulsion, and have decreased AprA binding. Expression of GrlH in *grlH¯* cells (*grlH¯/grlH*^*OE*^) rescues the phenotypes described above. These data indicate that AprA signaling may be mediated by GrlH in *D. discoideum*.

## IMPORTANCE

Little is known about how eukaryotic cells can count themselves and thus regulate the size of a tissue or density of cells. In addition, little is known about how eukaryotic cells can sense a repellant signal and move away from the source of the repellant, for instance, to organize the movement of cells in a developing embryo or to move immune cells out of a tissue. In this study, we found that a eukaryotic microbe uses G protein-coupled receptors to mediate both cell density sensing and chemorepulsion.

## INTRODUCTION

Chalones are secreted factors that inhibit the proliferation of the cells that secrete them (1, 2). For instance, melanocyte proliferation can be inhibited by an unknown secreted chalone, and when the crude chalone is injected under a melanoma, the melanoma cell proliferation ceases (3, 4). Despite their intrinsic interest and potential utility in controlling tumor growth, in most cases where chalone activity has been observed, the identity of the chalcone and the identity of the associated signal transduction pathway are unknown.

We previously identified two *Dictyostelium discoideum* chalones, AprA and CfaD. Both are secreted proteins that inhibit *D. discoideum* cell proliferation (5, 6). Cells lacking either AprA or CfaD show abnormally fast proliferation, and this phenotype can be rescued either by expressing AprA in *aprA¯* cells or CfaD in *cfaD¯* cells or by adding recombinant AprA or CfaD to the respective mutant strains (5–7). Both AprA and CfaD are necessary for proliferation inhibition, as recombinant AprA (rAprA) cannot rescue the *cfaD¯* phenotype and recombinant CfaD (rCfaD) cannot rescue the *aprA¯* phenotype (7, 8). Several components of the AprA-induced and/or CfaD-induced proliferation inhibition signaling pathway have been identified, including the ROCO kinase QkgA, the p21-activated kinase (PAK) family member PakD, the PTEN-like phosphatase CnrN, and the tumor suppressor RblA (9, 10, 11, 13, 18). Additionally, AprA functions to chemorepel *D. discoideum* cells, causing cells to move in a biased direction away from a source of AprA (12). QkgA, PakD, CnrN, and RblA are also involved in the AprA-induced-chemorepulsion signaling pathway (9–13, 18). Both AprA inhibition of proliferation and AprA induction of chemorepulsion require the G proteins Gβ and Gα subunit Gα8, and the binding of AprA to cell membrane is inhibited by GTPγS, suggesting that AprA functions through binding to a G protein-coupled receptor (GPCR) (8, 12).

*D. discoideum* has 61 genes encoding predicted proteins with sequence similarity to GPCRs (14, 15). At least 35 of the 61 genes are expressed in growing and proliferating (vegetative) cells (16). One GPCR mutant, the *crlA¯* strain, proliferates faster than the wild type and is insensitive to rAprA-induced proliferation inhibition (17). However, *crlA¯* cells bind AprA with kinetics similar to those of wild-type cells, suggesting that CrlA is not the AprA receptor (7).

In this study, we examined the sensitivity to AprA of eight additional GPCR mutants in an attempt to identify the AprA receptor. We identified four mutants that show insensitivity to AprA. Among these, we found that cells lacking GrlH show most of the phenotypes expected for cells lacking the AprA receptor, including reduced binding to AprA, suggesting that GrlH is an AprA receptor.

## RESULTS

### GPCR mutant screening suggests several AprA receptor candidates.

**FIG 1:**
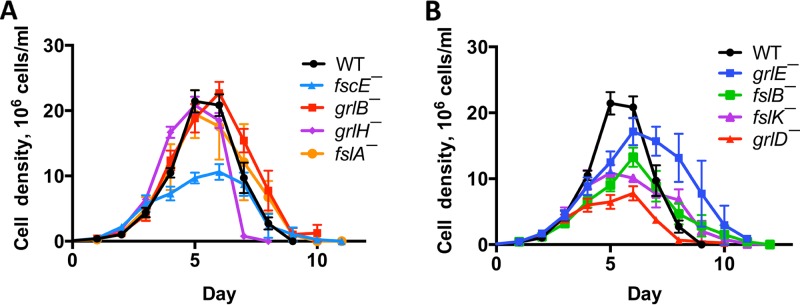
The effect of GPCR disruptions on proliferation. Log-phase cells were grown in liquid shaking culture starting at 1 × 10^5^ cells/ml and counted daily. WT, Ax2 wild type. For clarity, data from (A) fast-proliferation mutants and (B) normal-proliferation or slow-proliferation mutants were plotted separately. Values are means ± standard errors of the means (SEM); *n* ≥ 3 independent experiments.

AprA inhibition of proliferation and induction of chemorepulsion require the G protein subunits Gα8 and Gβ (8, 12), suggesting that AprA may signal through a G protein-coupled receptor. To identify the AprA receptor, we first determined whether any of an available set of mutants with insertions of a blasticidin resistance cassette in the coding region for a putative G protein-coupled receptor might have phenotypes similar to those of cells lacking AprA. Cells lacking AprA, Gα8, Gβ, or the AprA signal transduction components PakD, RblA, and QkgA exhibit faster proliferation and proliferate to a higher maximal density than wild-type cells (5, 8, 10, 11, 18). Examining the proliferation in a shaking culture of cells lacking putative G protein-coupled receptors, we observed that *grlH¯* cells showed significantly faster proliferation than wild-type cells, while *grlD¯* and *fscE¯* cells were slower to proliferate (Fig. 1) (Table 1). *grlH¯* cells also died faster after the stationary phase than wild-type cells (Fig. 1). None of the mutants proliferated to a higher density than the wild type, and some mutants proliferated to a lower maximal density (Fig. 1) (Table 1). Together, these results indicate that *grlH¯* cells, like *aprA¯* cells (5), have fast proliferation and die quickly after the stationary phase but do not have the *aprA¯* phenotype of proliferation to an abnormally high cell density.

**TABLE 1:** The effect of GPCR disruptions on doubling time and maximal density^a^

Cell type	Doubling time, h	Maximum observed cell density (10^6^ cells/ml)
WT	14.3 ± 0.2	21.9 ± 1.7
*grlB¯*	14.0 ± 0.4	23.4 ± 1.5
*grlD¯*	16.9 ± 0.8*	9.0 ± 0.6**
*grlE¯*	15.2 ± 0.5	18.8 ± 1.7
*grlH¯*	13.0 ± 0.1**	20.8 ± 1.3
*fslA¯*	14.8 ± 1.2	22.1 ± 4.2
*fslB¯*	16.7 ± 1.1	13.6 ± 1.3**
*fslK¯*	16.1 ± 1.3	13.7 ± 1.2**
*fscE¯*	15.8 ± 0.5*	12.0 ± 0.8**

a Doubling times and maximum cell densities were calculated using the data presented in Fig. 1. Values are means ± SEM; *n* ≥4 independent experiments. *, *P* < 0.05; **, *P* < 0.01 (compared to the wild type [*t* test]).

**FIG 2:**
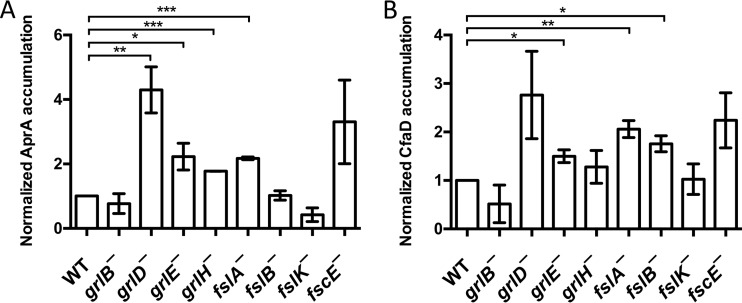
Accumulation of AprA and CfaD in GPCR mutants. Conditioned media from log-phase cells were analyzed for accumulated (A) AprA and (B) CfaD using anti-AprA and anti-CfaD antibodies. Quantifications (*n* = 3 independent experiments, means ± SEM) of Western blot bands, normalized to the band intensity for the Ax2 wild type (WT), are shown. *, *P* < 0.05; **, *P* < 0.01; ***, *P* < 0.001 (compared to the wild type [*t* test]).

One possible reason that a mutant *Dictyostelium* strain would exhibit abnormal proliferation is an abnormal accumulation of AprA or CfaD. To test this possibility, conditioned media from the putative G protein-coupled receptor mutants were assayed for AprA and CfaD. None of the mutants accumulated significantly less AprA or CfaD than wild-type cells (Fig. 2). Four mutants, including the *grlH¯* mutant, accumulated abnormally high levels of extracellular AprA. *grlH¯* cells accumulated normal levels of extracellular CfaD, and 3 other mutants accumulated abnormally high levels of extracellular CfaD. Together, these data indicate that the fast proliferation of *grlH¯* cells is not due to abnormally low extracellular levels of AprA or CfaD.

**FIG 3:**
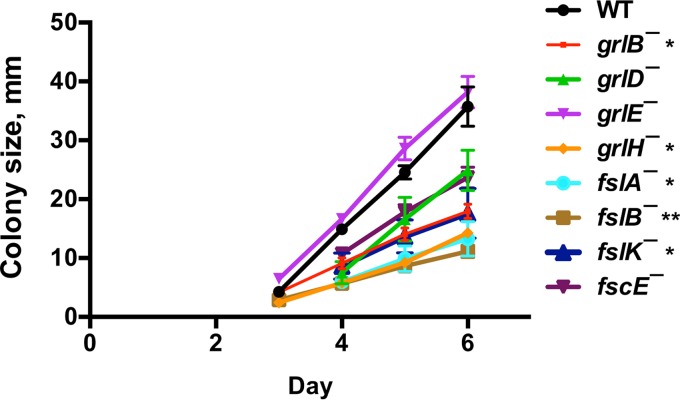
GPCR mutant colony expansion. Approximately 10 cells were plated onto agar plates spread with *K. aerogenes* bacteria. At least 3 colonies were measured daily per plate. After 6 days, individual wild-type colonies were indistinguishable from each other. Values are means ± SEM; *n* ≥ 3 independent experiments. The absence of error bars indicates that the SEM was less than the size of the marker. *, *P* < 0.05; **, *P* < 0.01 (for the change in colony size from day 4 to day 6 compared to the wild type [*t* test]).

**FIG 4:**
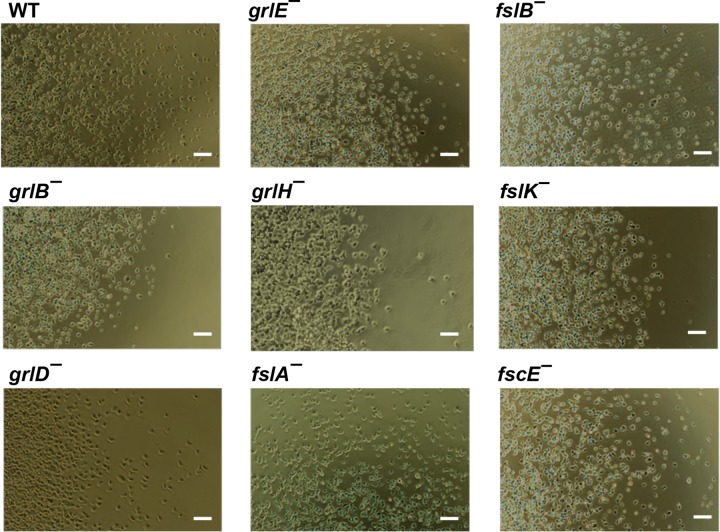
Colony edges of GPCR mutants. Colonies of cells were allowed to adhere to glass well slides. Fresh media were added to the wells, and cells were left overnight to spread. The edges of colonies were imaged with a 10× phase-contrast objective. The right colony edge is shown for all strains except the *fslA¯* mutant, where the top colony edge is shown. Images are representative of results of at least three independent experiments. Bars are 50 µm.

Possibly due to a decreased ability to cause cells at the edge of a colony to be repelled from the colony, *aprA¯*, *pakD¯*, *rblA¯*, and *qkgA¯* cells form abnormally small colonies starting from single cells on bacterial lawns (5, 10, 11, 18). The *grlB¯*, *grlH¯*, *fslA¯*, *fslB¯*, and *fslK*¯ cells also formed abnormally small colonies on bacterial lawns (Fig. 3). Whereas wild-type cells form colonies in submerged liquid culture with cells dispersed from the colony edges, *aprA¯*, *rblA¯*, and *qkgA¯* cells form colonies with well-defined edges and few dispersed cells (5, 10, 11). We found that *grlB¯*, *grlH¯*, and *fslK¯* cells also formed colonies with well-defined edges (Fig. 4). Together, these results indicate that *grlB¯*, *grlH¯*, and *fslK¯* cells all have the small colony on bacterial lawns and that the abnormally few cells dispersing from a colony in submerged liquid culture show phenotypes characteristic of cells lacking AprA or some of the AprA signal transduction pathway components.

Another characteristic of *aprA¯* cells is that they tend to be multinucleate; a population of *aprA¯* cells has fewer mononucleate cells and more cells with 2 nuclei or 3 or more nuclei than a population of wild-type cells (5). Cells lacking the AprA signal transduction pathway component Gα8, Gβ, or QkgA have similar multinucleate phenotypes (8, 10). We found that* grlD¯*, *fslA¯*, *fslB¯*, *fslK¯*, and *fscE¯* cells had fewer cells with a single nucleus, and more cells with two nuclei, than wild-type cells (Table 2). *fslB¯* and *fslK¯* cells also had more cells with three or more nuclei. These data indicate that, like cells lacking AprA, Gα8, Gβ, or QkgA, cells of several mutants lacking putative G protein-coupled receptors, but not *grlH¯* cells, tend to have more nuclei per cell than wild-type cells.

**TABLE 2:** The effect of GPCR disruptions on nuclei per cell^a^

Cell type	% of cells with *n* nuclei per cell:			No. of nuclei/ 100 cells
	1	2	3+	
WT	86 ± 2	14 ± 3	0.5 ± 0.2	116 ± 2
*grlB¯*	76 ± 3	22 ± 2	1.8 ± 0.6	126 ± 3*
*grlD¯*	62 ± 3***	34 ± 2**	3.7 ± 1.9	143 ± 5*
*grlE¯*	79 ± 4	20 ± 4	0.8 ± 0.6	122 ± 5
*grlH¯*	90 ± 2	9 ± 1	0.8 ± 0.2	109 ± 1
*fslA¯*	69 ± 4**	27 ± 3*	4.1 ± 2.2	136 ± 6*
*fslB¯*	71 ± 2**	27 ± 2*	2.4 ± 0.6*	132 ± 3*
*fslK¯*	49 ± 4***	41 ± 3***	9.4 ± 1.6**	167 ± 9**
*fscE¯*	71 ± 1***	29 ± 1**	0.9 ± 0.3	131 ± 2**

a Cells were stained with DAPI (4′,6-diamidino-2-phenylindole), the number of nuclei in cells was counted, and then the number of nuclei per 100 cells was calculated. Values are means ± SEM; *n* ≥ 3 independent experiments. *, *P* < 0.05; **, *P* < 0.01; ***, *P* < 0.001 (compared to the wild type [*t* test]).

**FIG 5:**
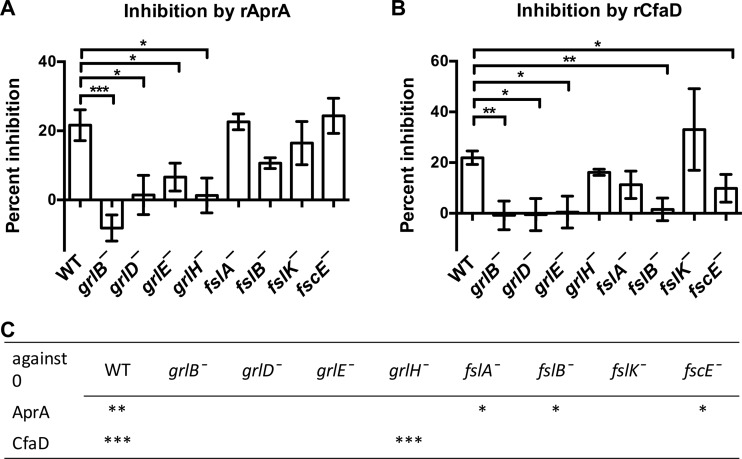
Sensitivities of GPCR mutants to inhibition of proliferation by rAprA or rCfaD. A 300-ng/ml volume of rAprA or rCfaD, or an equivalent volume of buffer, was added to cells in liquid shaking culture. After 16 h, cell densities were counted. (A and B) The percentage of inhibition of proliferation caused by rAprA (A) or by rCfaD (B) compared to the buffer control was calculated. (C) The significance of the difference between the effect represented by a value of 0 (no effect of AprA or CfaD) and the observed effect was calculated. Values are means ± SEM; *n* ≥ 3 independent experiments. *, *P* < 0.05; **, *P* < 0.01; ***, *P* < 0.001 (compared to the wild type [*t* test]).

Both AprA and CfaD inhibit the proliferation of *D. discoideum* cells (5, 6). To test the possibility that the fast proliferation of *grlH¯* cells may be due to reduced sensitivity either to AprA or to CfaD, we examined the ability of recombinant AprA (rAprA) and recombinant CfaD (rCfaD) to inhibit proliferation. Both proteins inhibited the proliferation of wild-type cells as previously observed (5, 6) (Fig. 5). As seen with the wild-type cells, AprA inhibited the proliferation of *fslA¯*, *fslB¯*, *and fscE¯* cells, and CfaD inhibited the proliferation of *grlH¯* cells. Cells lacking GrlB, GrlD, GrlE, or GrlH were insensitive to rAprA, and cells lacking GrlB, GrlD, GrlE, FslB, or FscE were insensitive to rCfaD (Fig. 5). Together, these results suggest that, surprisingly, multiple receptors are required for the ability of rAprA or rCfaD to inhibit proliferation.

**FIG 6:**
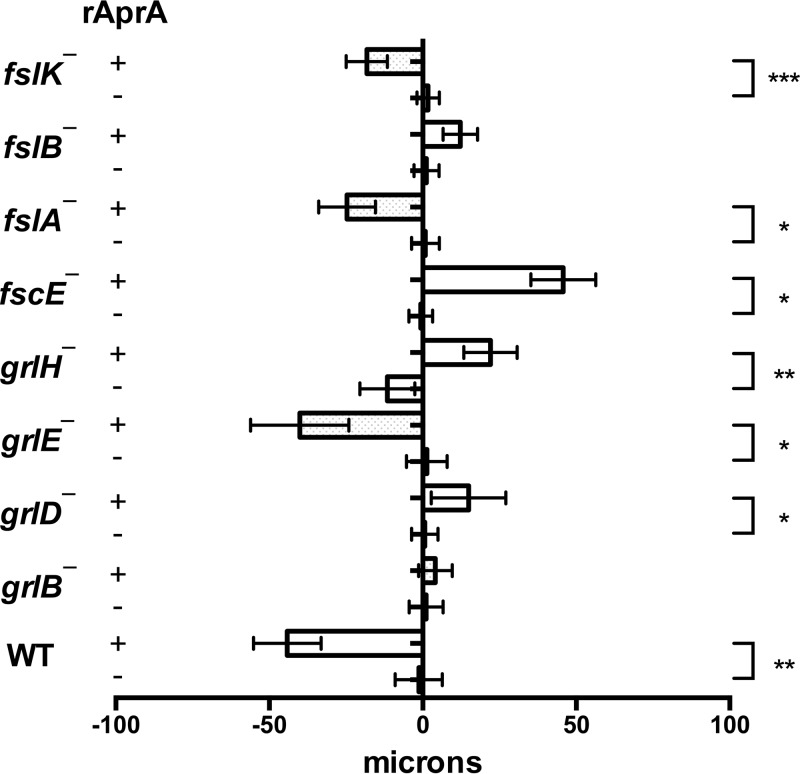
The effect of rAprA on chemorepulsion in GPCR mutants. rAprA or an equivalent volume of buffer was added to one side of an Insall chamber. Videomicroscopy was used to visualize the movement of cells, which were then manually tracked. The migration distance along the gradient over the course of 60 min was measured. Values are means ± SEM; *n* ≥ 3 independent experiments. Bars to the left of the vertical line at the center of the figure represent cells moving away from the source. *, *P* < 0.05; **, *P* < 0.01; ***, *P* < 0.001 (*t* test).

AprA, but not CfaD, is a chemorepellent for *D. discoideum* cells (12). We used an Insall chamber assay (12, 19) to determine if any of the putative G protein-coupled receptor mutants are insensitive to rAprA-induced chemorepulsion. As we previously observed, wild-type cells moved in a biased direction away from rAprA (12) (Fig. 6). *grlE¯*, *fslA¯*, and *fslK¯* cells also moved away from rAprA (Fig. 6). For unknown reasons, *grlD¯*, *grlH¯*, and *fscE¯* cells moved toward the source of rAprA. *grlB¯* and *fslB¯* cells were insensitive to AprA-induced chemorepulsion (Fig. 6). These data suggest that, with respect to chemorepulsion from a source of rAprA, several GPCR mutants are insensitive to AprA.

**FIG 7:**
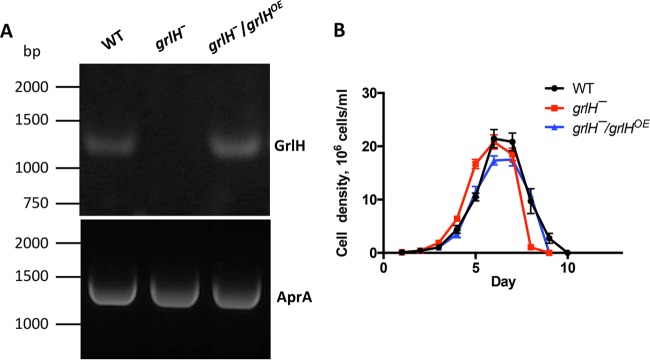
The effect of GrlH on proliferation. (A) *grlH* expression (top panel) was confirmed by RT-PCR, using RT-PCR with primers for AprA as a loading control (bottom panel). (B) Cell proliferation was measured as described for Fig. 1. Values are means ± SEM; *n* ≥ 3 independent experiments.

None of the GPCR mutants showed phenotypes matching all of the phenotypes of *AprA*¯ cells or the phenotypes of mutants that are insensitive to AprA. Only *grlB¯*, *grlD¯*, and *grlH¯* cells had defects in both AprA-induced proliferation inhibition and AprA-induced chemorepulsion. These results suggest that there may be more than one AprA receptor in *D. discoideum*. Among those three mutant strains, only the *grlH¯* cells were insensitive to AprA-induced proliferation inhibition but not to CfaD-induced proliferation inhibition. Similarly to *aprA¯* cells, *grlH¯* cells showed a higher death rate after the stationary phase, a lower colony expansion rate on bacterial lawns, tighter colony edges in submerged culture, and a shorter doubling time than wild-type cells. These data suggest that GrlH may be at least one of the AprA receptors. To test the hypothesis that GrlH is an AprA receptor, we expressed *grlH* under the control of the *actin15* promoter in *grlH¯* cells to make the rescue strain *grlH¯/grlH*^*OE*^. *grlH* expression was observed in wild-type and *grlH¯/grlH*^*OE*^ cells but not in *grlH¯* cells (Fig. 7A). Expression of *grlH* in the *grlH¯* cells increased the doubling time to a level similar to that of wild-type cells (Fig. 7B) (Table 3), suggesting that the fast proliferation of *grlH¯* cells is due to lack of GrlH. The *grlH¯/grlH*^*OE*^ cells had a lower maximal cell density than wild-type cells (Table 3), suggesting that GrlH may regulate cell density.

**TABLE 3:** The effect of GrlH on doubling time and stationary density^a^

Cell type	Doubling time, h	Maximum observed cell density (10^6^ cells/ml)
WT	14.3 + 0.2	21.9 + 1.7
*grlH¯*	13.0 + 0.1***	20.8 + 1.3
*grlH¯/grlH^OE^*	14.1 + 0.3	17.5 + 1.2*

a Doubling times and maximum cell densities were calculated using the data in Fig. 7B. Values are means ± SEM; *n* ≥ 4 independent experiments. *, *P* < 0.05; ***, *P* < 0.001 (compared to the wild type [one-way ANOVA, Tukey’s test]). The difference between the doubling time of the *grlH¯* mutant and that of the *grlH¯/grlH*^*OE*^ mutant was significant with *P* < 0.01; all other differences were not statistically significant (one-way ANOVA, Tukey’s test).

**FIG 8:**
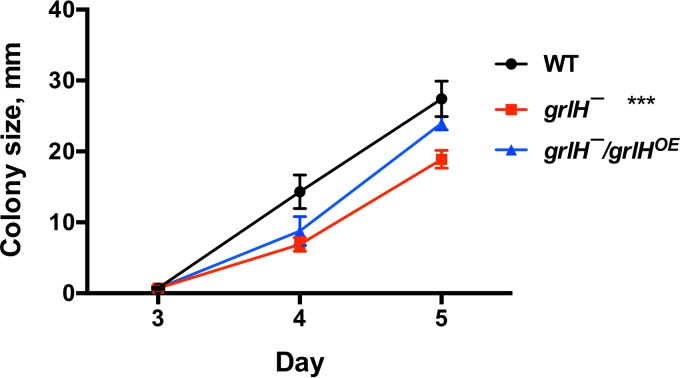
*grlH¯* and *grlH¯ grlH*^*OE*^ colony expansion. Approximately 10 cells were plated onto plates containing *E. coli* bacteria. At least 3 colonies were measured per plate daily. Values are means ± SEM; *n* ≥ 3 independent experiments. The absence of error bars indicates that the SEM was less than the size of the marker. ***, *P* < 0.001 (for the change in colony size from day 3 to day 5 compared to the wild type [one-way ANOVA, Tukey’s test]). The difference between the *grlH¯* mutant and the *grlH¯/grlHOE* mutant was significant with a *P* value of <0.01, and there was no significant difference between WT and the *grlH¯/grlH*^*OE*^ mutant (one-way ANOVA, Tukey’s test).

**FIG 9:**
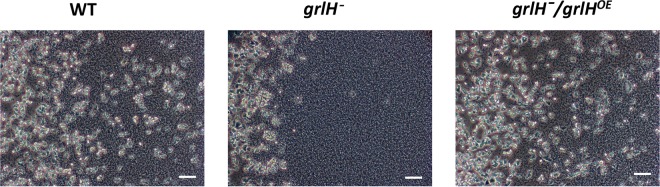
Colony edge formation of the *grlH¯* and *grlH¯/grlH*^*OE*^ mutants. Colonies of cells were allowed to adhere to glass well slides. Fresh SM media with *E. coli* was added to the wells, and the cells were left overnight to spread. The edges of colonies were imaged with a 10× phase-contrast objective. The right colony edge is shown for all strains. Images are representative of results of at least three independent experiments. Bars are 50 µm.

**FIG 10:**
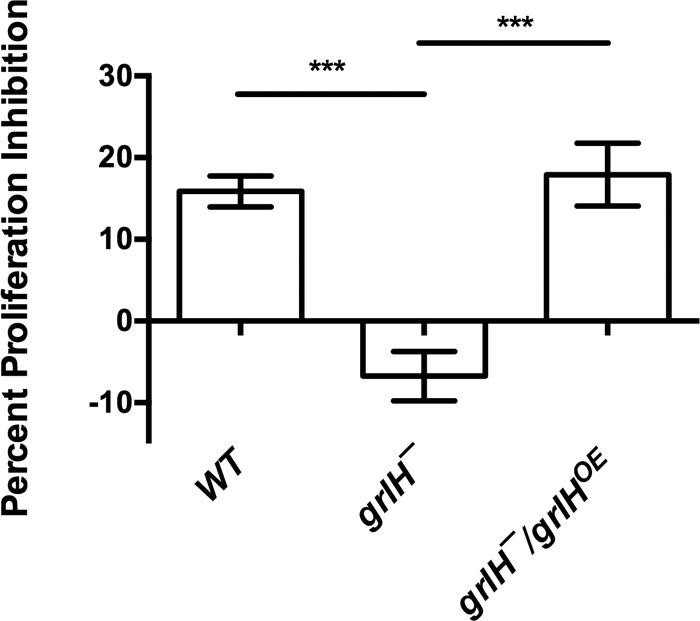
The effect of GrlH on AprA-induced proliferation inhibition. The effect of AprA on proliferation was measured as described for Fig. 5. Values are means ± SEM; *n* ≥ 3 independent experiments. ***, *P* < 0.001 (one-way ANOVA, Tukey’s test). There is no significant difference between WT and the *grlH¯/grlH*^*OE*^ mutant (one-way ANOVA, Tukey’s test).

**FIG 11:**
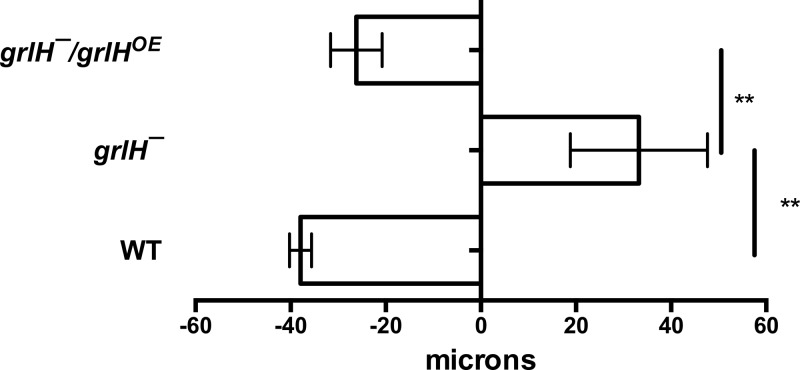
The effect of GrlH on AprA-induced chemorepulsion. rAprA or an equivalent volume of buffer was added to one side of an Insall chamber. Videomicroscopy and tracking were then done as described for Fig. 5. Values are means ± SEM; *n* ≥ 3 independent experiments. **, *P* < 0.01 (one-way ANOVA, Tukey’s test). There is no significant difference between WT and the *grlH¯/grlH*^*OE*^ mutant (one-way ANOVA, Tukey’s test).

Expression of *grlH* in *grlH¯* cells also rescued the *aprA¯-*like phenotypes of *grlH¯* cells such as a low colony expansion rate on bacterial lawns (Fig. 8) and tight colony edges (Fig. 9). Expression of *grlH* in *grlH¯* cells also restored the ability of *grlH¯* cells to decrease proliferation in the presence of AprA (Fig. 10) and to be repelled by a source of rAprA (Fig. 11). These data indicate that the slower colony expansion, the tighter colony edges, and the abnormal sensitivity to rAprA of *grlH¯* cells are due to the lack of GrlH.

**FIG 12:**
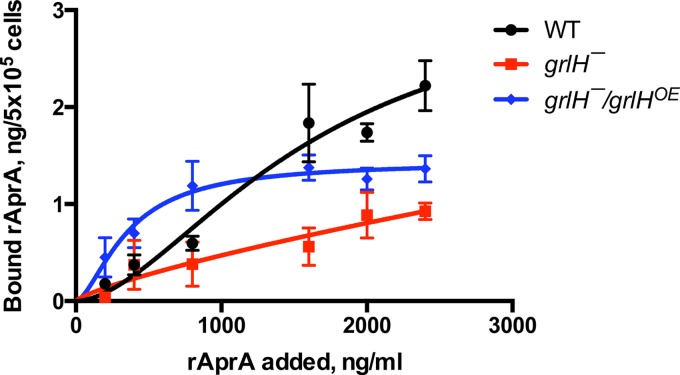
The effect of GrlH on AprA binding to cells. Cells of the indicated strains were incubated with the indicated concentrations of myc-tagged rAprA at 4°C. After 15 min, cells were collected by centrifugation, resuspended in ice-cold HL5, and collected, and the bound rAprA was measured by Western blotting (with known amounts of myc-rAprA on each blot for quantitation of bands), staining for the myc tag. Values represent means ± SEM; *n* ≥ 3 independent experiments. The lines represent curves fitting to a one-site binding model.

We previously observed that wild-type cells bind rAprA with a *B*_max_ of 3.1 ± 0.4 ng/5 × 10^5^ cells and a dissociation constant (*K*_*d*_) of 160 ± 50 ng/ml (7). To directly test the hypothesis that GrlH is required for cells to bind rAprA, we did rAprA binding assays on cells. *grlH¯* cells showed a decreased level of rAprA binding compared to wild-type cells, and *grlH¯/grlH*^*OE*^ cells showed a partially rescued level of rAprA binding (Fig. 12). We measured *B*_max_ and *K*_*d*_ values for rAprA binding to wild-type cells of 3.3 ± 2.0 ng/5 × 10^5^ cells and 1,600 ± 1,200 ng/ml, respectively. For unknown reasons, the *K*_*d*_ value that we measured was much higher than what we had previously observed. The *B*_max_ and *K*_*d*_ values for rAprA binding to *grlH¯/grlH*^*OE*^ cells were 1.4 ± 0.2 ng/5 × 10^5^ cells and 360 ± 100 ng/ml, respectively. The *grlH¯* cells did not show saturable binding of rAprA. These data suggest that loss of GrlH decreases rAprA binding to cells and that binding of rAprA can be restored by expression of *grlH* in the *grlH¯* cells.

## DISCUSSION

We previously found that AprA requires G proteins to inhibit proliferation and to induce chemorepulsion, suggesting that AprA is a ligand for a GPCR (8). In this report, we show that, compared to wild-type cells and similarly to mutants which are insensitive to AprA, cells lacking the GPCR GrlH have a lower doubling time, a lower colony expansion rate, and tighter colony edges (10–12, 18). An important caveat is that, in addition to AprA, many other factors could contribute to the doubling time of cells, the colony expansion rate, and the morphology of the colony edge. In addition, cells lacking GrlH are insensitive to rAprA-induced proliferation inhibition and chemorepulsion and show reduced binding to rAprA compared to wild-type cells. Expressing GrlH in the *grlH¯* cells rescued the phenotypes described above. Together, these data suggest that GrlH is a receptor for AprA. Although AprA is glycosylated, the effects of recombinant AprA produced in bacteria, and thus not significantly glycosylated, mimic the effects of endogenous AprA (5–7), and we observed an apparent binding of recombinant AprA to GrlH, suggesting that GrlH binds to a nonglycosylated feature of AprA.

Based on function and amino acid sequence, GPCRs can be classified into the following families: family 1, containing an β-adrenergic, odorant receptor and light receptors; family 2, containing secretins; family 3, containing metabotropic glutamate/GABA_B_ receptors; family 4, containing pheromone receptors; and family 5, containing frizzled/smoothened receptors (20, 21). In humans, more than 1,000 genes encode GPCRs. In *Dictyostelium*, 61 genes encode putative GPCRs (14, 15). One gene, *lrlA*, belongs to family 2; 17 genes (*grlA* to *grlH* and *grlJ* to *grlR*) belong to family 3; 25 genes (*fslA* to *fslH*, *fslJ* to *fslQ*, *fscA* to *fscH*, and *fscJ*) belong to family 5; 12 genes (*cAR1* to *cAR4* and *CrlA* to *CrlH*) belong to a unique cAR/Crl (cAMP receptor/cAMP receptor like) family; 1 gene encodes a protein similar to orphan vertebrate protein GPR89; and 5 genes encode proteins with similarity to human transmembrane protein 145 (15, 17, 22, 23). CAR1 to CAR4 are cAMP receptors, GrlB and GrlE are γ-aminobutyric acid receptors, and GrlL (fAR1) is a folate receptor (8, 24–28). Like GrlB, GrlE, and GrlL, GrlH is a family 3 receptor.

Several GPCR mutants other than the *grlH¯* mutant were insensitive to rAprA-induced proliferation inhibition or chemorepulsion or both. *grlB¯*, *grlD¯*, and *grlE¯* cells were insensitive to rAprA-induced proliferation inhibition, and *grlB¯*, *grlD¯*, *fslB¯*, and *fscE¯* cells were insensitive to rAprA-induced chemorepulsion. One possible explanation for those findings is that two or more receptors exist for AprA, as cells lacking GrlH did not show completely abolished rAprA binding. In support of the idea that multiple receptors might sense AprA, cells lacking GrlH were attracted to a source of recombinant AprA, indicating the presence of a non-GrlH receptor that mediates chemoattraction to AprA. If this unknown receptor is closely related to GrlH, it may be among the 17 family 3 receptors that are not GrlB, GrlE, GrlH, or GrlL. Since the recombinant AprA is not glycosylated, this unknown receptor appears to sense a nonglycosylated feature of AprA. Another possible explanation is that these receptors are activated by a different signal and that this signaling is necessary for AprA signaling. For instance, cells lacking CfaD are insensitive to rAprA-induced proliferation inhibition and chemorepulsion (7, 12). Unlike *grlH¯* cells, *grlB¯*, *grlD¯*, and *fslB¯* cells were insensitive to rCfaD-induced proliferation inhibition. It is possible that the disruption of GrlB, GrlD, or FslB interrupts the CfaD signaling pathway such that *grlB¯*, *grlD¯*, and *fslB¯* cells cannot response properly to AprA. In addition to CfaD, there may be other factors that are necessary for AprA and CfaD signaling. Together, these results indicate that GrlH is a receptor for AprA, that there may be more than one AprA receptor, and that multiple receptors and, presumably, their associated signaling pathways regulate AprA signaling.

## MATERIALS AND METHODS

### Cell culture and strains.

*grlB¯* (DBS0350074), *grlD¯* (DBS0350227), *grlE¯* (DBS0350075), *grlH¯* (DBS0350226), *fslA¯* (DBS0350228), *fslB¯* (DBS0350230), *fslK¯* (DBS0350229), and *fscE¯* (DBS0350232) cells were generated by homologous recombination in the wild-type Ax2 background using the vector pLPBLP (29). The 5′ homologous region and the 3′ homologous region for each gene were amplified from the genomic DNA and directionally cloned into pLPBLP. Primers used to amplify these regions for each GPCR gene are listed in Table S1 in the supplemental material. The resulting construct was linearized with NotI, and 2 μg of linearized DNA was electroporated into 5 × 10^6^ cells (29). The transformed cells were grown with 10 µg/ml blasticidin S for 10 days, diluted, and then plated on a *Klebsiella aerogenes* lawn. Clones were isolated 5 days later, and successful gene disruption in clones was confirmed by PCR of genomic DNA using one primer inside the blasticidin resistance cassette and one primer in the genomic DNA outside the homologous region used for the knockout construct (Table S2) (30). At least 2 different clones were isolated, and phenotypes were confirmed. Parental Ax2 cells and mutants were grown as previously described (31) in SM medium with *E. coli* or in HL5 medium (Formedium Ltd., Norwich, England).

10.1128/mBio.02443-17.1 TABLE S1  Primers used for gene disruptions. Underlined nucleotides indicate restriction sites. Download TABLE S1, DOCX file, 0.1 MB. Copyright © 2018 Tang et al. 2018 Tang et al. This content is distributed under the terms of the Creative Commons Attribution 4.0 International license.

10.1128/mBio.02443-17.2 TABLE S2  Primers used for verifying gene disruptions. A forward GPCR primer was paired with a reverse plPBLP primer and vice versa. Download TABLE S2, DOCX file, 0.05 MB. Copyright © 2018 Tang et al. 2018 Tang et al. This content is distributed under the terms of the Creative Commons Attribution 4.0 International license.

### Assays.

Proliferation in shaking culture, proliferation inhibition, chemotaxis, nucleus staining, colony expansion, spore count, and spore viability assays were done following the methods described in references 10 and 13 except that *Escherichia coli* was also used for colony expansion. Colony edge imaging was done following the method described in reference 10 except that for GPCR mutant screening, 200 µl of HL5 medium without bacteria was added to each well. AprA and CfaD accumulation assays were done as previously described (6, 7). Preparation of recombinant His-tagged AprA and CfaD was done following the methods described in references 6 and 7. rAprA binding to cells was measured as previously described (7), with the exception that 0, 200, 400, 800, 1,600, 2,000, or 2,400 ng/ml rAprA was added to cells and incubated with the cells for 15 min at 4°C, and biotinylated mitochondrial 3-methylcrotonyl-CoA carboxylase α (MCCC1) was used as a gel loading control (32).

### *grlH* expression.

To construct a *grlH* expression vector, total RNA from vegetative Ax2 cells was isolated using an R1054 RNA prep kit (Zymo Research, Irvine, CA) and then a cDNA library was generated using this RNA as a template with a K1651 cDNA synthesis kit (Thermo Fisher, Carlsbad, CA). PCR was done using this cDNA with primers 5′-CGCGGATCCATGAAAAATATTTTAAAAATT-3′ and 5′-CCGCTCGAGTTAATTATTATTTTCTGAATCATTG-3′ to generate a DNA fragment containing the *grlH* coding region. After digestion with BamHI and XhoI (NEB, Ipswich, MA), the PCR product was ligated into the corresponding sites of pDXA-3D (33) to produce expression plasmid pDXA-3D-grlH. To construct the *grlH¯/grlH*^*OE*^ strain, *grlH¯* cells were transformed with pDXA-3D-grlH by electroporation following the method described in reference 34. The expression of *grlH* was verified by reverse transcription-PCR (RT-PCR) with primers 5′-GCTTCCGAAAGAGCCACC-3′ and 5′-CAATAAAGCCGCAGTGGT-3′, with RNA extraction and cDNA synthesis done as described above. For a loading control, RT-PCR was done with primers for AprA (5′-CCCAAGCTTACTCCATTGGATGATTATGTC-3′ and 5′-CCGCTCGAGTAAAGTTGCAGTTGAACTAGCACTATCACC-3′).

### Statistics.

Statistical analyses performed with *t* tests and one-way analysis of variance (ANOVA) performed with the appropriate posttest and curve fits were done using Prism (GraphPad, San Diego, CA). Significance was defined as a *P* value of <0.05.
