# Supplementary material for: An Autocrine Proliferation Repressor Regulates Dictyostelium discoideum Proliferation and Chemorepulsion Using the G Protein-Coupled Receptor GrlH
Source: mBio. 2018 Feb 13;9(1):e02443-17. doi: 10.1128/mBio.02443-17 (PMC5821085; doi:10.1128/mBio.02443-17)
Supplement: TABLE S1 [file mbo001183715st1.docx]

**Table S1: Primers used for receptor knockouts.**

| Primer name | Primer sequence (5’-3’)^a^ |
| --- | --- |
| *fscE5-F-KpnI* | GGGGTACCTACAACAGATAGACAAAGTG |
| *fscE5-R-HindIII* | CCCAAGCTTGCATCAATGTAACCATACTG |
| *fscE3-F-PstI* | AACTGCAGGGATTTGAAATATCAAGTTGG |
| *fscE3-R-SpeI* | GGACTAGTAGGTTCATTTGATTGCTCAG |
| *fslA5-F-SalI* | GCGTCGACAGACCATGTAGAGAATCATG |
| *fslA5-R-HindIII* | CCCAAGCTTGCAACACATCTTGAATCATG |
| *fslA3-F-PstI* | AACTGCAGGTTGGATTATGAGTAATTCAG |
| *fslA3-R-SpeI* | GGACTAGTAAATCATCATCATCACCACC |
| *fslB5-F-KpnI* | GGGGTACCATATGGAGCAGGATTAGTTG |
| *fslB5-R-HindIII* | CCCAAGCTTTTGATGCCATTTCTTCTCTG |
| *fslB3-F-PstI* | AACTGCAGGCAGTTCAATCAGATGTTTC |
| *fslB3-R-NotI* | TTGCGGCCGCTGAAGTTGGATTTGTACCAC |
| *fslH5-F-SalI* | GCGTCGACATGTGTGCAATGATGTTTCC |
| *fslH5-R-HindIII* | CCCAAGCTTTACACCTGAAACTGACATTC |
| *fslH3-F-PstI* | AACTGCAGGATAGAGTATGTGTTGCATC |
| *fslH3-R-SpeI* | GGACTAGTTAGGGAATTGCTGAATCTTC |
| *fslK5-F-KpnI* | GGGGTACCGTATAGAATGCACATGACTG |
| *fslK5-R-HindIII* | CCCAAGCTTGTTGTGCTTCTGTATATGTG |
| *fslK3-F-BamHI* | CGGGATCCAAGAGTTTGTTTGGATACTTG |
| *fslK3-R-SpeI* | GGACTAGTCCAGATTCAATCCAAACTAC |
| *grlB5-F-KpnI* | GGGGTACCAAGTGGTGACTTTTCAGATC |
| *grlB5-R-SalI* | GCGTCGACTCCGAGGTCAATGTACATTG |
| *grlB3-F-PstI* | AACTGCAGGAAGGAACTTGTAGAGCAAG |
| *grlB3-R-BamHI* | CGGGATCCCATCTTCAGTATCACTACTG |
| *grlD5-F-SalI* | GCGTCGACTGTTCAAGTCAAGATCATGC |
| *grlD5-R-HindIII* | CCCAAGCTTGGGATAATCTTGGTGTTCTC |
| *grlD3-F-PstI* | AACTGCAGGGTAATGATGGGTTTAGTAG |
| *grlD3-R-SpeI* | GAACATATCACTAGTTCCAC |
| *grlE5-F-KpnI* | GGGGTACCCAGAAGTTGTTAAACCAAACCC |
| *grlE5-R-HindIII* | CCCAAGCTTGATTACGAAGTTCAGTTCTAAC |
| *grlE3-F-BamHI* | CGGGATCCTACAGGTGATAGATTGTATGG |
| *grlE3-R-NotI* | ATTTGCGGCCGCAAAGATTGGTTCAGCCAATGG |
| *grlH5-F-SalI* | GCGTCGACGAATCAAGGTAGAATTGGAG |
| *grlH5-R-HindIII* | CCCAAGCTTATCAGTGCTAACACCATAAC |
| *grlH3-F-PstI* | AACTGCAGAACTCCATCAATGAGATCAG |
| *grlH3-R-BamHI* | CGGGATCCGAGGTAACATTAGCAACATC |
