# Supplementary material for: An Autocrine Proliferation Repressor Regulates Dictyostelium discoideum Proliferation and Chemorepulsion Using the G Protein-Coupled Receptor GrlH
Source: mBio. 2018 Feb 13;9(1):e02443-17. doi: 10.1128/mBio.02443-17 (PMC5821085; doi:10.1128/mBio.02443-17)
Supplement: TABLE S2 [file mbo001183715st2.docx]

**Table S2: Primers used for verifying gene disruption.**

| Primer name | Primer sequence (5’-3’) |
| --- | --- |
| fscE-KO-R | GTTTAGAATCATCAATACCTG |
| fslA-KO-F | CCAAACTTGTGTTCCAATTTC |
| fslB-KO-F | CATTTGGATTAAATTTACCAGATGG |
| fslK-KO-R | GTTGTACCTACTGTTAAAACTATAC |
| grlB-KO-R | CTTCCAGCAATTTGTTCATC |
| grlD-KO-R | CACTTGAACCTTTACTTGTAC |
| grlE-KO-R | CTGAAGTACATTGAAGTTGAGC |
| grlH-KO-F | GTCTTGATGGAGATGGAAAAC |
| PLPBLP-R | CTAGAGGATCTATAACTTCG |
| PLPBLP-F | GATGTAAAACAGCCAAAGAG |
